# Multimodal artificial intelligence in glioma management: integrating neuroimaging and hematologic biomarkers for precision oncology

**DOI:** 10.3389/fonc.2026.1812518

**Published:** 2026-04-20

**Authors:** Rafail C. Christodoulou, Platon S. Papageorgiou, Daniel Eller, Evros Vassiliou, Sokratis G. Papageorgiou, Elena E. Solomou, Michalis F. Georgiou

**Affiliations:** 1 Division of Neuroimaging and Neurointervention, Department of Radiology, Stanford University, Stanford, CA, United States; 2 Department of Medicine, National and Kapodistrian University of Athens, Athens, Greece; 3 Department of Biological Sciences, Kean University, Union, NJ, United States; 4 1st Department of Neurology, Medical School, National and Kapodistrian University of Athens, Eginition Hospital, Athens, Greece; 5 Internal Medicine-Hematology, University of Patras Medical School, Rion, Greece; 6 Department of Radiology, University of Miami, Miami, FL, United States

**Keywords:** artificial intelligence, gliomas, hematologic biomarkers, multimodal data fusion, neuro oncology, neuroimaging, precision oncology, pseudoprogression (PsP)

## Abstract

**Background:**

Gliomas are biologically heterogeneous primary brain tumors that remain challenging to diagnose, prognosticate, and monitor noninvasively, owing to marked intratumoral heterogeneity, treatment-related imaging changes, and limited accessibility of tissue biomarkers. Despite advances in molecular classification, clinical decision-making still relies heavily on neuroimaging, highlighting the need for integrative, data-driven approaches.

**Objective:**

This narrative review examines how artificial intelligence (AI) can integrate multimodal neuroimaging with hematologic and other liquid biomarkers to support clinical decision-making in glioma management.

**Content:**

We synthesize recent advances in machine learning (ML) and deep learning (DL) applied to MRI and PET for glioma detection, segmentation, molecular phenotype inference, and outcome prediction. We review both segmentation-based and segmentation-free modeling paradigms, highlighting their respective assumptions, advantages, and limitations. Advanced imaging techniques, including diffusion (DWI, DTI) and perfusion imaging, MR spectroscopy, and metabolic and amino acid PET, are discussed as sources of biologically specific signals that extend beyond conventional structural imaging. We further examine blood-derived biomarkers, such as inflammatory and immune mediators, circulating nucleic acids, and extracellular vesicle cargo, which provide complementary insights into tumor–host interactions and enable longitudinal assessment. Emerging generative and systems-level modeling approaches are also reviewed in the context of multimodal data integration and clinical application.

**Conclusion:**

Multimodal AI has the potential to integrate spatial imaging phenotypes with systemic biological signals to improve noninvasive diagnosis, molecular risk stratification, and treatment monitoring in gliomas. Translation to clinical practice will depend on appropriate methodological design choices, standardized workflows, rigorous external validation, uncertainty-aware decision support, and continuous performance monitoring in real-world settings.

## 1 Introduction

Gliomas are a diverse group of primary brain tumors with considerable variation in their clinical behavior, molecular characteristics, prognosis, and how they respond to treatment (1, 2). Their presentation often includes neurological, cognitive, and behavioral impairments that can significantly affect quality of life. Despite significant progress in neuroimaging, molecular classification, and chemotherapy regimens, radiotherapy planning, overall survival rates for patients with high-grade gliomas, especially glioblastoma (GBM), have improved only modestly in recent decades, remaining below 16 months (3).

Overall survival rates in GBM remain low due to intratumoral heterogeneity, infiltration, and early treatment resistance (4). Specifically, the 10-year overall survival rate in a large cohort of GBM patients was estimated at approximately 0.71% (5). On the other hand, low-grade gliomas, such as astrocytomas and oligodendrogliomas, have survival rates averaging 7 years and often exceeding 10 years in selected cases (6, 7).

Unfortunately, the survivors can face long-term neurocognitive, endocrine, and psychosocial issues, highlighting the need for better diagnostics and monitoring (8). Overall, poor survival in adult gliomas and survivors’ long-term emotional and physical burden represent a critical unmet need. This highlights the need for more accurate, non-invasive diagnostic tools and disease monitoring, as well as improved risk stratification, thereby driving interest in AI-driven, multimodal biomarker approaches (9). Clinicians often encounter significant challenges in diagnosing, predicting outcomes, and monitoring patients with gliomas. Typically, the diagnostic process involves neuroimaging, followed by neurosurgical tissue sampling for histopathological and molecular testing. Surgical biopsies or resections offer definitive diagnosis but are invasive, pose risks, and may only sample part of a heterogeneous tumor (10). Although incorporating molecular markers, such as IDH mutations or 1p/19q codeletion, into glioma classification has improved tumor classification under the latest WHO classification (2, 11), reliable non-invasive biomarkers for diagnosis or early relapse detection remain scarce. The blood–brain barrier greatly limits the release of tumor-derived molecules into the bloodstream, resulting in low levels of circulating tumor DNA and other markers (12). Consequently, traditional blood tests are of limited use in diagnosing gliomas, with ongoing monitoring mainly depending on repeated imaging scans. For example, a retrospective study emphasized the significance of systemic markers like the neutrophil-to-lymphocyte ratio (NLR) and the systemic immune-inflammation index (SII) in distinguishing genuine tumor progression from pseudoprogression in gliomas. This process usually takes months of follow-up using imaging criteria, reinforcing this approach (13). The complexities of brain anatomy, challenges in tumor accessibility, significant heterogeneity within and between tumors, and a scarcity of prognostic biomarkers all impede effective clinical decision-making in brain tumor treatment (14). These challenges drive the development of new tools to enhance diagnosis and prediction in neuro-oncology.

AI, including ML and DL, quickly detects complex patterns in large datasets that humans may not be able to identify.ML models are trained on quantitative data extracted from images, called radiomics, and analyze these data to predict biomarkers, treatment response, or survival (2, 15). The integration of explainability provides valuable insights into the importance of features for each prediction and links them to tumoral biomarkers (16). DL could serve as a non-invasive biopsy to confirm tumor grade, identify biomarkers, and distinguish treatment responses (17, 18). Meanwhile, DL can segment gliomas with high accuracy to assist radiotherapy planning and deliver the prescribed dose directly to the malignant tumor (19). Additionally, AI models can reduce MRI scan times by improving image quality and reconstruction, which is particularly beneficial for patients with brain tumors, for whom prolonged MRI examinations are often poorly tolerated and associated with increased motion artifacts. Reducing acquisition time can improve image quality, decrease motion-related degradation, and substantially enhance patient comfort and compliance (20). AI models that integrate liquid biomarkers, such as circulating tumor DNA, cells, and blood molecules, with imaging improve diagnostic accuracy. AI models integrate this data, enhancing precision and risk assessment and providing a comprehensive view of tumor biology by merging imaging with biomarker data (21).

This narrative review summarizes recent progress in AI for glioma management, emphasizing the combination of multimodal neuroimaging and blood biomarkers. It critically evaluates segmentation-based and segmentation-free modeling approaches, advanced and metabolic imaging methods, generative techniques, and systems-level frameworks relevant to gliomas. The review discusses clinical applications, design considerations, and translational hurdles in the use of multimodal AI to enhance noninvasive diagnosis, molecular profiling, prognosis, and treatment monitoring in patients with glioma.

## 2 Literature search strategy

A comprehensive literature search was conducted by an academic medical librarian to identify relevant studies on artificial intelligence–based multimodal approaches in neuro-oncology. The search strategy was developed in accordance with established recommendations for transparent reporting in narrative reviews, including the SANRA guidelines (22).

Targeted searches were conducted in PubMed/MEDLINE, Embase, and Scopus from January 2000 to October 2025 using combinations of controlled vocabulary and keyword terms related to brain tumors, neuroimaging, artificial intelligence, machine learning, and blood-derived biomarkers. Studies were included if they addressed clinically relevant AI applications in neuro-oncology and excluded if they lacked a clinical context or an original methodological contribution.

In addition to structured database searches, targeted supplementary references were incorporated to address emerging methodological areas and identified conceptual gaps. These studies were identified through expert knowledge of the field, citation tracking of key reviews and seminal articles, and focused manual screening of recent high-impact peer-reviewed publications. This approach prioritizes conceptual breadth and clinical relevance over exhaustiveness and may introduce selection bias, a limitation acknowledged. Narrative review methodology does not require exhaustive or reproducible searches, but it does emphasize transparent reporting of how the literature was identified and selected (22, 23). Consequently, it is acceptable to use a structured search as the foundation and then incorporate targeted supplementary references based on expert knowledge, citation tracking, and reviewer feedback, provided that this process is clearly described and selection bias acknowledged (24).

To minimize potential selection bias typical of narrative reviews, several strategies were employed. The study selection process was guided by predefined conceptual priorities, such as clinical relevance, methodological rigor, and representation of major AI approaches in neuro-oncology, rather than focusing only on highly positive-performing results. When multiple studies addressed similar tasks, preference was given to those with external validation, multicenter data, or transparent methodological reporting. Citation tracking helped gather both supporting and contrasting evidence, ensuring a balanced discussion. Additionally, recent peer-reviewed literature was prioritized to reflect the current state of the field. Although formal systematic review procedures were not followed, these measures aimed to improve transparency, prevent selective emphasis, and ensure a balanced conceptual overview of the review.

Full database-specific search strategies and Boolean logic are provided in the Appendix.

## 3 Discussion

### 3.1 Artificial intelligence in conventional neurooncology imaging

AI applications have become increasingly promising in recent years for interpreting neurooncology imaging. Early efforts in this domain applied ML to analyze handcrafted, quantified imaging features, laying the groundwork for modern radiomic-based approaches. The extraction of these features from neuroimages and their analysis by ML models provided insights into tumor tissue characteristics, perfusion-related signal patterns, shape-intensity distributions, and spatial heterogeneity that cannot be obtained through visual assessment alone (25). Traditional ML models, such as support vector machines, random forests, and logistic regression, have demonstrated promising results in differentiating among brain tumor types, providing molecular profiling information, and predicting survival outcomes in patients with brain tumors (26).

Radiomics-based ML is beneficial because it enables the integration of clinical data and tumor biomarkers, such as MGMT or IDH, in gliomas to achieve comprehensive prediction. However, radiomics pipelines are sensitive to variations in imaging acquisition, preprocessing, and feature selection, limiting generalizability. A recent literature review confirmed these concerns while examining the role of radiomics in distinguishing progression, recurrence, pseudoprogression, and radiation necrosis in GBM. Although several studies report superior performance compared to radiologists’ evaluations and achieve high performance metrics, the results should be interpreted with caution due to small sample sizes, varying preprocessing methods, and the lack of external validation (27).

Similarly, a radiomics-based study by Yu et al. (2017) aimed to predict IDH1 status in grade II gliomas and included 110 patients, from whom 671 features from T2-FLAIR were extracted; it reported an accuracy of 0.80 and was validated in an independent cohort (28). The authors noted that the metrics can be improved by incorporating multiple imaging modalities, a point supported by Li et al. (2018), who conducted a multicenter study exploring the role of a multiregional multimodal radiomics model and achieved an accuracy of 0.96 in predicting IDH status (29). Another crucial biomarker in gliomas that radiomics have been applied to predict is MGMT. While there is no specific MRI appearance of MGMT tumors, Xi et al. (2017) reported an accuracy of 0.82 in a study of 98 GBM patients using T1-enhanced images, further supporting the idea that radiomics may be an essential biomarker for guiding preoperative management of GBM (30).

A highly correlated biomarker with these is ATRX, as multiple radiomics studies have tried to predict it with promising results. For instance, Mora et al. (2023) reported an accuracy of 0.74 and noted that T2WI may be the most effective sequence for biomarker prediction (31). Building on this, a meta-analysis by C.Y.C. et al. (2025) evaluated radiomics studies that predict IDH and ATRX. It reported pooled AUCs of 0.84 and 0.95, based on 11 studies involving gliomas grades I-IV (32).

DL studies have demonstrated that convolutional neural networks (CNNs) are effective in neurooncology, particularly for biomarker prediction, segmentation, and classification. To support reproducible development and benchmarking of brain tumor segmentation methods, the multimodal Brain Tumor Segmentation Benchmark (BraTS) has become pivotal in neuro-oncology AI research (33). As a large, multi-institutional challenge, BraTS provides standardized multimodal MRI datasets with expert annotations, thereby spurring rapid advances in glioma segmentation and outcome prediction. To support clinical and scientific applications, Kofler et al. developed the BraTS toolkit, a modular system that integrates preprocessing, automated segmentation, and consensus fusion of multiple advanced algorithms, thereby reducing technical barriers and enhancing robustness across centers (34). Consequently, the BraTS ecosystem is now essential for validating segmentation pipelines and remains a key reference for supervised neuro-oncology imaging research.

Multitasking in novel DL frameworks can predict tumor grading and biomarkers without requiring tumor segmentation (35). This method, known as segmentation-free supervised learning, avoids the need for tumor delineation by enabling the model to analyze the entire brain or a broadly defined region. It uses attention mechanisms or global feature learning to detect patterns that are prognostically or biologically significant without relying on pixel-level annotations. Li et al. (2022) demonstrated that glioma survival predictions from whole-brain MRI could be made accurately using a deep attention network, yielding AUCs ranging from 0.74 to 0.94 at 6–48 months across various centers, without requiring tumor segmentation (36). Huang et al (2023) demonstrated another example of unsupervised tumor delineation using a feature-aware method that recognizes brain tumor morphology in MRI without manual annotations. Using fDDFT, geometric DL, and graph-based features, it identifies lesion boundaries from image data, achieving median Dice scores above 0.75 across various datasets (37). This demonstrates that meaningful imaging and accurate tumor boundaries can be achieved entirely without supervision, decreasing dependence on expensive expert segmentations and enabling scalable, segmentation-free workflows. Additionally, recent transformer-based unsupervised anomaly detection models learn typical patterns from healthy brain MRI scans and identify tumors using reconstruction-error maps, achieving a detection rate of 89.4%, thereby enabling tumor segmentation across diverse tumor types without manual labels (38). Collectively, these approaches highlight how segmentation-free supervised learning can facilitate scalable, label-efficient tumor localization, each employing different assumptions and computational strategies.

Segmentation plays a key role in radiomics-based studies, where precise delineation is required before extracting features to predict tumor biomarkers, survival outcomes, or to differentiate treatment responses in gliomas. For instance, Chen et al (2021) proposed a hybrid CNN-radiomics model for PTEN mutation prediction where the features were extracted following an automated segmentation using DL and fused with the handcrafted radiomics features. Importantly, this model achieved superior performance when used alone, with a reported AUC of 0.91, compared with the CNN (0.84) and the radiomics model (0.83) (39). Likewise, Decuyper et al. (2020) developed an automated pipeline that simultaneously segments and predicts glioma grade, IDH status, and 1p19q codeletion, achieving accuracies of 94%, 86%, and 87% on an externally validated data dataset (40). In radiomics, the use of automated segmentation may be efficient and convenient, but it reduces delineation accuracy, which can affect feature stability and, in turn, model performance and prediction accuracy due to propagation error (41). In contrast, automated DL models can facilitate greater deployability and scalability. Segmentation is also vital in radiation therapy because accurately defining tumor boundaries helps limit the dose to cancerous cells. As a result, various CNN architectures have been employed, with U-Net being the most prevalent. For example, Amri et al. (2025) developed an automated U-Net that achieved high segmentation accuracy, with a Dice score of 92.54% and an IoU of 90.42%, while maintaining a processing time of 1.5 seconds per image (42). Similarly, in biomarker prediction, several CNN approaches have attempted to predict MGMT, with varying levels of accuracy reported. For instance, Chang et al. (2018) designed a CNN that can simultaneously predict IDH, 1p/19q codeletion, and MGMT with accuracies of 0.94, 0.92, and 0.83, respectively (43). Lastly, CNNs have shown promising results in distinguishing between GBM and brain metastasis, a standard diagnostic dilemma. Specifically, a 3D CNN developed by Bathla et al. (2024) improved radiologists’ accuracy; a model using the T1 enhancement mask achieved an AUC of 0.93 (44).

Overall, segmentation should be seen as a task-specific design decision rather than a universal necessity. It remains essential for applications requiring accurate spatial localization, like radiotherapy planning and volumetric analysis. However, for tasks such as survival modeling and biomarker prediction, where reducing annotation effort is crucial, segmentation-free approaches may be more suitable, as they can improve robustness and ease deployment.

### 3.2 Artificial intelligence in advanced neurooncology imaging

While conventional structural MRI provides essential anatomical information, advanced neuroimaging techniques offer complementary insights into tumor cellularity, perfusion, and metabolism (45, 46). The integration of AI with these advanced imaging modalities has substantially expanded the ability to characterize brain tumors beyond morphology alone, enabling more biologically informed assessments of diagnosis, prognosis, and treatment (**Table 1**) (14, 54, 55).

**Table 1:** AI and advanced neurooncology imaging: the clinical contributions of each modality.

Modality	What it captures	High-yield clinical task(s)	Typical AI/feature strategy	Example biomarkers/outputs	Reference
Diffusion MRI (DWI/ADC)	Cellularity/microstructure	Pre-op molecular profiling and grading support	ML combine conventional and advanced MRI features	IDH, MGMT, ATRX, EGFR. Improved performance when diffusion was added	(46)
Diffusion Tensor Imaging (DTI)	White-matter microstructural integrity and tumor infiltration along fiber tracts	Assessment of glioma invasiveness, surgical,radiotherapy planning and molecular prediction	Radiomics based ML on tensor measures	Glioma Grade, OS and Biomarker Prediction	(47–49)
MR Spectroscopy (MRS/MRSI)	*In vivo* metabolites	Tumor grading	ML/DL on spectroscopy regions	Discriminative metabolic signatures; key metabolites highlighted (e.g., NAA, Glu, Gln)	(50)
Hyperpolarized MRI (HP-MRI)	Real-time metabolic flux (e.g., pyruvate→lactate)	Early response assessment; progression/recurrence tumor biology	DL multimodal fusion of HP-MRI and conventional MRI.	Predict early response/relapse while expands beyond single ratio metrics via learned metabolic patterns	(51)
Amino-acid PET (FET PET)	Tumor amino-acid transport/metabolism (higher specificity than FDG in brain)	Tumor delineation grading and post-treatment evaluation	ML models with static and dynamic PET metrics	Better definition of active tumor and differentiation of treatment-related changes vs progression	(52)
Hybrid PET/MRI (FET PET + mpMRI)	Spatially aligned metabolism and structure/perfusion/cellularity	Surgery/RT planning; multi-modal diagnosis	Combined feature spaces; radiomics based ML	Identifies metabolically active tumor beyond contrast enhancement margins. Superior performance compare to solely MRI	(53)

Diffusion-based imaging, including diffusion-weighted imaging (DWI) and apparent diffusion coefficient (ADC) mapping, reflects tumor cellularity and structure and has been increasingly incorporated into AI-driven radiomic models. Studies combining diffusion-derived features with conventional MRI radiomics have demonstrated improved performance in predicting key glioma molecular biomarkers, including IDH mutation, MGMT promoter methylation, ATRX loss, and EGFR alterations (46, 56). Zhao et al. (2025) proposed that DWI b-values can effectively predict IDH and 1p/19q codeletion status in gliomas, with an AUC of 0.79 (56). Additionally, incorporating DWI sequences into conventional MRI can improve the predictive performance of preoperative biomarker models (46). Similarly, perfusion metrics, such as rCBF, have been linked to glioma genetic profiles and can predict IDH status specificity (57). Findings such as these demonstrate that diffusion imaging captures microstructural heterogeneity that is not fully represented by anatomical sequences alone.

While diffusion imaging is primarily associated with diffusion-weighted MRI, diffusion tensor imaging (DTI) provides critical additional microstructural insights into white matter integrity, tumor grade, infiltration, and survival prediction (47, 58, 59). This information is especially valuable for surgical planning and prognosis in gliomas. DTI metrics, such as fractional anisotropy and mean diffusivity, are often combined with structural MRI and integrated into ML and multimodal AI systems rather than used alone (48). For example, Yuan et al. (2024) showed that DTI radiomics provides valuable information for IDH prediction in gliomas, achieving an AUC of 0.847 when combined with conventional radiomics, highlighting its important supplementary role (49).

Magnetic resonance spectroscopy (MRS) provides *in vivo* metabolic information by quantifying tumor-associated metabolites and has shown promise for improving diagnostic specificity when combined with AI (50). ML approaches applied to MRS and spectroscopic imaging have enabled the identification of discriminative metabolic signatures for glioma grading and classification, with metabolites such as N-acetyl aspartate, glutamate, and glutamine emerging as biologically relevant markers. Several ML models developed by Fidrous et al. (2024) have been used to identify the key areas contributing to glioma grading discrimination, achieving accuracies ranging from 0.87, depending on the number of regions involved (50).

Metabolic imaging approaches provide additional insights into tumor biology by detecting dynamic changes in cancer metabolism that often precede structural alterations. Notably, hyperpolarized magnetic resonance imaging (HP-MRI) enables real-time, *in vivo* visualization of metabolic fluxes, such as the pyruvate-to-lactate conversion, and serves as a sensitive indicator of tumor growth, treatment response, and recurrence, particularly in aggressive cancers such as GBM (51). When combined with AI, HP-MRI data can be integrated with traditional anatomical MRI to identify metabolic patterns associated with early treatment response and disease progression (51). Furthermore, AI-based frameworks that connect *in vivo* HP-MRI with ex vivo nuclear magnetic resonance metabolomics and immunohistochemical analysis have shown promise in predicting tissue-specific metabolic biomarkers solely from imaging data. This broadens the potential for metabolic characterization beyond simple ratio measures (60). These results highlight AI’s potential to integrate *in vivo* metabolic imaging with ex vivo metabolomic profiles, enabling earlier detection of tumor changes and more accurate, metabolism-focused monitoring.

Positron emission tomography (PET), especially when combined with MRI, has become a powerful tool for assessing brain tumor metabolism and improving diagnosis beyond traditional imaging (52). Amino acid PET tracers, like 18F-fluoroethyltyrosine (FET), outperform 18F-FDG by targeting tumor-specific amino acid transport and reducing background uptake in normal tissue. A meta-analysis reinforces that FET-PET outperformed FDG-PET for isolated tumor diagnosis, whereas in glioma grading, performance was similar (61). Static and dynamic FET PET measures, such as tumor-to-brain ratios and time–activity curves, help delineate tumors, grade them, and distinguish them from treatment effects, including pseudoprogression and radiation necrosis (45). When combined with MRI in hybrid PET/MRI systems, FET PET accurately identifies metabolically active tumor areas beyond contrast-enhancing margins, aiding surgical and radiotherapy planning. Combining FET- PET with perfusion MRI, diffusion imaging, and MR spectroscopy provides synergistic diagnostic benefits that exceed those of any single modality. ML applied to PET/MRI data further improves tumor classification and molecular profiling, including the prediction of IDH mutation status (53), highlighting PET’s valuable role in advanced neuro-oncologic imaging.

AI-driven neuroimaging techniques, such as diffusion imaging, spectroscopy, and metabolomics, provide a comprehensive view of tumor biology, revealing details of cellularity, metabolism, and heterogeneity. These improve diagnosis, profiling, and prognosis and integrate multimodal frameworks that combine imaging and liquid biomarkers, thereby advancing precision neuro-oncology.

### 3.3 Generative modeling in neuro-oncology imaging

Generative modeling has become a vital component of neuro-oncology AI, helping to address issues such as limited data, incomplete multimodal imaging, and inefficient acquisition (62). A recent study by Moon et al. showed that diffusion-based generative augmentation can significantly improve MRI-based prediction of IDH mutation status in gliomas by generating large, high-quality T1-weighted and FLAIR images. This approach yielded consistent performance improvements across cohorts and even surpassed the accuracy of expert neuroradiologists (63). In addition to data augmentation, generative models enable meaningful image synthesis and modality translation. For example, Miao et al. used a conditional GAN to generate post-operative contrast-enhanced MRI from pre-operative MRI and post-operative CT, thereby aiding the evaluation of resection extent in GBM when standard post-operative MRI is unavailable (64). Generative and self-supervised reconstruction models also aid MRI denoising and accelerate image reconstruction, producing diagnostic-quality images from undersampled or low-signal scans. This reduces scan time and enhances patient comfort, which is especially important for neuro-oncology patients who may experience pain, cognitive challenges, or difficulty remaining still during lengthy examinations (65). The rapid development of medical image synthesis architectures from CNNs and GANs to transformers and diffusion models and their increasing use in MRI-only radiotherapy planning, PET/MRI workflows, cross-modality harmonization, and faster imaging acquisition (66). Overall, these studies show that generative AI can support multimodal neuro-oncology workflows but face challenges before clinical adoption. High computational costs and infrastructure requirements, particularly for diffusion-based and transformer models that require substantial GPU resources and long training times, limit their use in resource-poor settings. Model validation and generalizability are issues, as synthetic images may not preserve subtle disease features across scanners, institutions, or populations (67). There’s also concern over hallucinated or spurious features that can impact tumor assessment and treatment planning (68). Quantitative evaluation methods are not standardized, and image similarity metrics don’t always reflect clinical relevance (69). Emerging validation frameworks seek to tackle these issues through layered evaluation methods. These methods include expert radiologist during tests to verify clinical realism, downstream task validation, in which synthetic data are assessed for their impact on diagnostic or prognostic model accuracy, and biological fidelity checks to assess whether synthetic images retain important radiologic–pathologic relationships. Other strategies include uncertainty quantification, testing for domain shifts across institutions, and multimodal consistency analysis to confirm that synthetic data preserve meaningful connections among imaging modalities (70–72). These validation techniques are increasingly regarded as essential steps for the safe clinical adoption of generative AI. Finally, regulatory approval, transparency, and clinician trust remain unresolved, underscoring the need for uncertainty-aware models, external validation, and the integration of outputs as decision support rather than replacements (73).

### 3.4 Mathematical neuro-oncology and systems biology

Mathematical neuro-oncology offers a mechanistic complement to data-driven AI models by explicitly simulating tumor growth, invasion, and treatment response. Foundational studies have introduced reaction–diffusion and proliferation–invasion models that link MRI-visible tumor size to biological processes such as cellular proliferation and infiltration, thereby enabling patient-specific predictions of tumor growth and recurrence (74). Subsequent studies confirmed that parameters derived from routine MRI using these models can inform prognosis and survival, underscoring their clinical significance (75).

More broadly, systems biology frameworks have been shown to capture glioma invasion dynamics and tumor–microenvironment interactions that are difficult to infer from imaging alone (76). Increasingly, these mechanistic models are viewed as synergistic with modern AI approaches, as mathematical models can provide biologically grounded priors or interpretable parameters for AI pipelines, while AI methods can assist in estimating model parameters from large-scale imaging data. Such hybrid strategies may improve robustness, interpretability, and clinical trust in neuro-oncologic decision-support systems. For example, parameters from mechanistic tumor growth models, such as tumor cell proliferation rates and infiltration coefficients derived from reaction–diffusion models, can serve as meaningful biological features in ML models aimed at predicting survival or assessing treatment response. Conversely, AI techniques such as DL feature extraction can directly estimate these parameters from MRI scans by recognizing imaging patterns associated with tumor invasion and spatial heterogeneity. These hybrid methods enable mechanistic models to provide biologically interpretable variables, while AI enhances the accuracy of parameter estimation and predictive performance through model-optimization algorithms. This two-way integration offers a promising approach for merging biological insight with the robust predictive power of data-driven AI models.

### 3.5 Hematologic and liquid biomarkers in brain tumors

Liquid biomarkers are increasingly recognized as a valuable complement to neuroimaging because they can reveal tumor–host biology, including inflammation, immune suppression, and metabolism. They also enable ongoing monitoring with less invasiveness than repeated tissue biopsies (77).

In gliomas, immune responses and cytokine signals are significant, given their substantial impact on the tumor immune microenvironment and prognosis. Recent studies show that AI-driven imaging techniques can link to these blood-derived pathways by noninvasively estimating levels of immune mediators detectable in blood. For instance, radiomics-based ML models have been able to predict IL18 levels and to differentiate overall survival in low-grade glioma, suggesting a potential imaging-based surrogate for immune activity that could be combined with blood tests to improve risk and overall survival assessment (78). A recent radiomics ML study that integrated genomics and imaging identified IL7R expression as a significant predictor of poor prognosis in high-grade gliomas. The validated radiomics models, which used survival data, effectively classified IL7R-associated risk groups without requiring invasive procedures. Higher IL7R risk scores were associated with reduced survival and with features such as increased microglial infiltration, a higher mutation burden, and activation of oncogenic pathways (79). Similarly, radiomics has been employed to predict chemokine signatures, such as CCL2, in high-grade glioma, reinforcing the concept that liquid-accessible inflammatory pathways can be inferred and used for prognosis-focused modeling (80). Immunophenotyping studies also highlight important immune niches in GBM, including immunosuppressive environments in tumors adjacent to the lateral ventricle and increased numbers of immunosuppressed immune cells (81, 82). Notably, analyses of histogram-based preoperative contrast-enhanced MRI have identified links between quantitative imaging features and tumor-infiltrating CD8^+^ T cells, which are crucial for better overall survival in GBM. Specific intensity distribution metrics, particularly those that reflect intratumoral heterogeneity, show moderate effectiveness in distinguishing tumors by CD8^+^ T-cell density (83). These results reinforce the idea that noninvasive imaging features can act as proxies for the immune microenvironment, complementing blood and tissue biomarkers and supporting comprehensive immuno-radiologic risk evaluation.

Beyond soluble cytokines, blood extracellular vesicles (EVs) have emerged as an increasingly investigated liquid biopsy substrate in brain tumors, as they carry tumor- and microenvironment-derived molecular cargo into the peripheral circulation (77, 84). Recent work using SWATH-MS–based proteomics has demonstrated the feasibility of profiling blood EVs for glioma surveillance, generating high-dimensional protein signatures that can be coupled with computational modeling for disease risk prediction and longitudinal monitoring.

Within a multimodal AI framework, EV-based assays are well aligned with precision neuro-oncology, providing systemic and serial biological readouts that complement MRI and PET imaging. AI methods are particularly well-suited to handling the complexity of EV proteomic data, enabling the identification of patterns associated with tumor presence, progression, and treatment response (**Table 2**).

**Table 2:** AI and clinical value of biomarkers.

Biomarker class	Example(s) and what it reflects clinically	AI and imaging intersection	Typical clinical value	Reference
Soluble inflammatory/immune mediators	Cytokine/chemokine signaling captures tumor–host immune activity and systemic inflammation.	Radiomics can act as a noninvasive surrogate by learning imaging patterns linked to immune mediator(cytocines) levels.	Risk stratification, prognosis support, and longitudinal immune state tracking alongside imaging.	(78)
IL18 (LGG context)	Immune activity signal associated with survival stratification.	MRI radiomics ML can predict IL18 expression and separate survival groups.	Adds biologic context to imaging-based risk with potential in combined imaging and blood risk modeling.	(78)
IL7R (HGG context)	Prognostic immune-related marker, higher risk scores track worse outcomes and correlate with immune and tumor mutation landscape.	Radiomics ML can classify IL7R-associated risk groups noninvasively and reveal genetic information	Prognosis modeling and biologically informed stratification without repeat tissue sampling.	(79)
Chemokine signatures	CCL2 as an inflammatory axis in high-grade glioma.	Radiomics models can predict CCL2 expression, supporting liquid information inference from imaging.	Prognosis-focused modeling and immune–microenvironment phenotyping.	(80)
Tumor immune microenvironment phenotypes	Immunological information(e.g., immunosuppressed patterns in specific anatomic contexts) linked to outcomes.	Imaging-derived features may approximate immune infiltration patterns and suppressive environments.	Supports immuno-radiologic risk evaluation when tissue is limited or heterogeneous.	(81, 82)
Imaging proxy of intratumoral CD8^+^ T-cell density	CD8^+^ T cells correlate with better OS T1CE histogram heterogeneity metrics predict CD8^+^ from imaging characteristics.	Histogram/radiomics features can estimate immune infiltration, complementing blood/tissue biomarkers.	Noninvasive immune phenotyping that can contextualize immunotherapy relevance and prognosis.	(83)
Extracellular vesicles (EVs)	Tumor and microenvironment cargo in blood	SWATH-MS EV proteomics yields high-dimensional signatures suited to ML for disease risk prediction and monitoring.	Serial surveillance and treatment-response tracking that complements MRI/PET.	(84)

EV contain diverse molecular cargo, including tumor-derived proteins, messenger RNAs, microRNAs, long non-coding RNAs, lipids, and DNA fragments that reflect both tumor biology and tumor–microenvironment interactions (85). Proteomic profiling of EVs has revealed signatures associated with glioma invasion, immune modulation, angiogenesis, and treatment resistance, highlighting their potential role in tumor surveillance and longitudinal monitoring (84). Similarly, microRNAs derived from EVs, including miR-21, miR-221, and miR-222, have been associated with glioma progression and could serve as minimally invasive biomarkers (86, 87). The complex, high-dimensional structure of EV molecular data, with its network-like relationships, makes it particularly well-suited to advanced AI methods beyond traditional machine learning. Newer architectures like graph neural networks and multimodal representation learning are particularly useful because they can model biological interaction networks, pathway relationships, and cross-omic dependencies (88, 89). These methods have the potential to facilitate more biologically informed modeling of tumor signaling pathways and enhance integration with imaging-derived phenotypes, marking a significant future step for precision medicine neuro-oncology.

### 3.6 Artificial intelligence multimodal integration of neuroimaging and hematologic biomarkers

**Figure 1:**
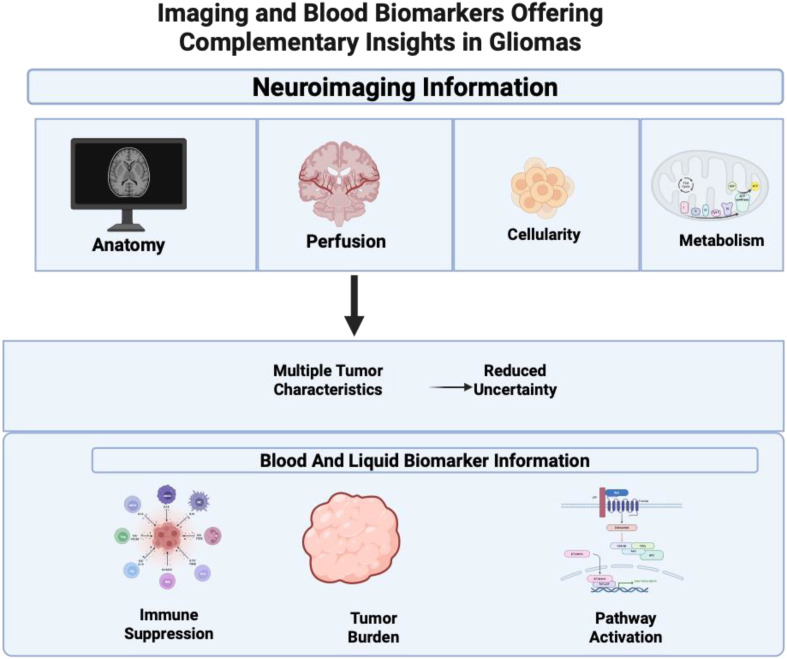
Complementary roles of neuroimaging and blood-based biomarkers in gliomas. Created in BioRender. Christodoulou, R. (2026) https://BioRender.com/o7agtsc. Neuroimaging captures spatial and functional tumor characteristics, including anatomy, perfusion, cellularity, and metabolism, whereas blood and cerebrospinal fluid biomarkers reflect systemic processes such as immune suppression, tumor burden, and pathway activation. Integrating these complementary data sources provides a more comprehensive tumor profile and reduces diagnostic and prognostic uncertainty in glioma management.

Multimodal AI aims to improve neuro-oncologic decision-making by combining neuroimaging with systemic or tumor-derived blood signals. Each modality captures distinct disease features: imaging provides spatial details on tumor anatomy, perfusion, cellularity, and metabolism, while blood biomarkers can indicate immune suppression, inflammation, tumor burden, and pathway-level biology (**Figure 1**). Together, these signals help reduce diagnostic uncertainty, enhance risk assessment, and enable better longitudinal tracking than a single modality.

In clinical practice, multimodal fusion most benefits three main tasks: tumor differentiation, noninvasive molecular profiling, and prognosis prediction. For diagnosis, combined models unify imaging features such as enhancement patterns, diffusion/perfusion signatures, and amino-acid PET uptake with blood profiles that reflect immune and microenvironmental status. This improves discrimination when tumors have overlapping imaging features. To gain molecular insights, imaging-based predictions of key markers can be enhanced by incorporating systemic biomarkers associated with tumor immunity (e.g., IL18/IL7R risk signatures or chemokine-related inflammation), thereby providing detailed patient profiles without invasive procedures. Regarding prognosis, merging imaging diversity with systemic immune or metabolic signals yields stronger survival predictions and earlier detection of aggressive disease courses.

Additionally, a particularly impactful application is treatment monitoring. Post-therapy MRI can often be inconclusive, and distinguishing actual progression from pseudoprogression is vital. In specific cases, multimodal AI can combine serial imaging data with concurrent trends in liquid biomarkers, such as EV proteins, inflammatory mediators, or cfDNA (90). Concordant increases, such as enhanced PET signals (91), alongside worsening systemic biomarkers, may increase confidence in true progression. Conversely, discordant or improving blood markers with stable or improving imaging suggest treatment effects. Fusion approaches for multimodal AI models include early and late fusion, combined via shared layers or attention (92). Intermediate and late fusion are often preferred in clinical settings as they handle diverse data types and missing modalities while clearly attributing contributions of each modality.

### 3.7 Clinical adaptability

**Figure 2:**
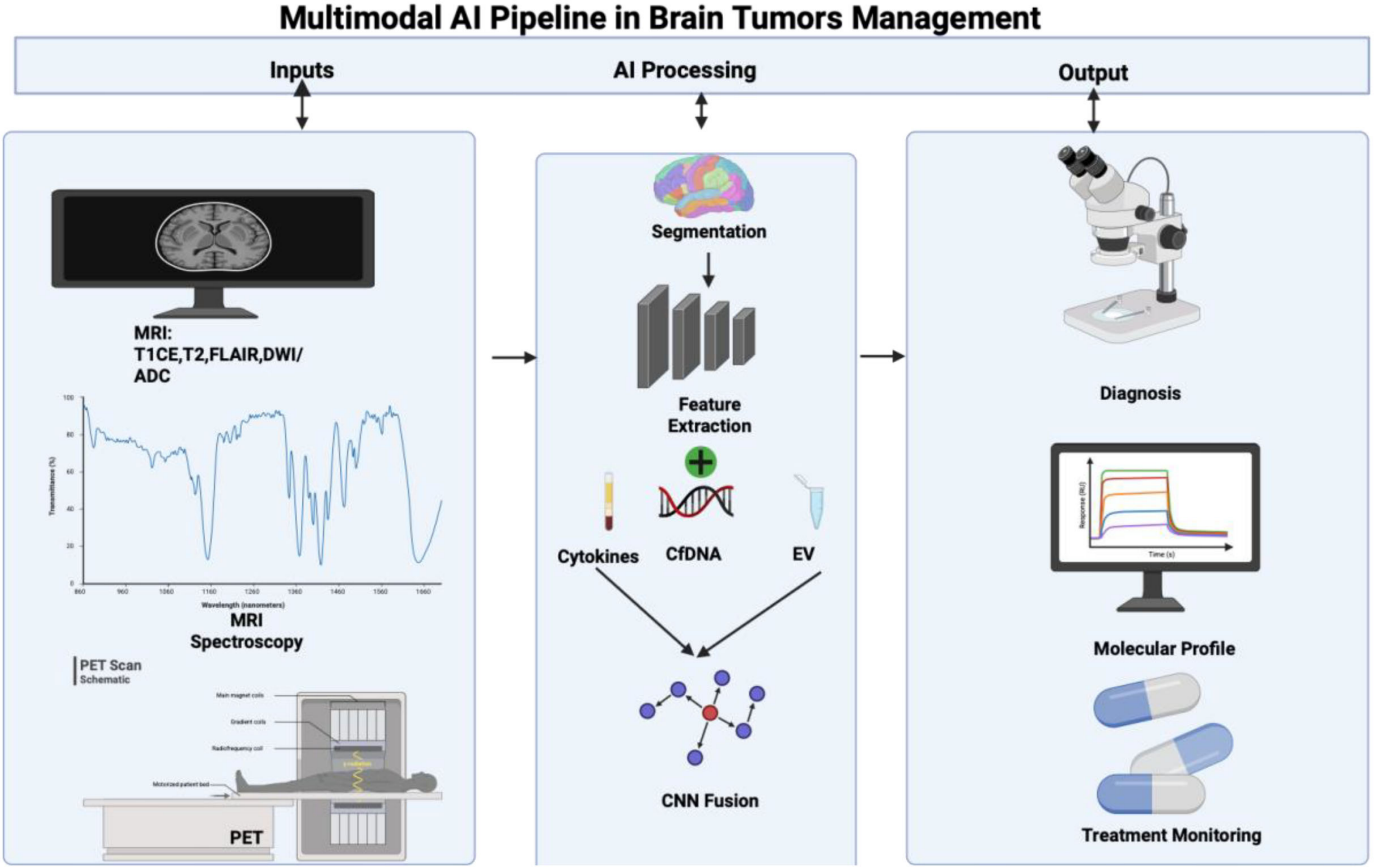
Multimodal AI pipeline for brain tumor management. Created in BioRender. Christodoulou, R. (2026) https://BioRender.com/ilhaiuy. Multiparametric neuroimaging (MRI, MR Spectroscopy, PET) and hematologic biomarkers (cytokines, cfDNA, extracellular vesicles) are integrated through AI-based segmentation, feature extraction, and deep learning fusion. The resulting models support diagnosis, molecular profiling, and treatment monitoring, enabling clinically informed decision-making in neuro-oncology.

Clinical adaptability depends more on whether a multimodal model integrates well with real-world workflows and remains reliable across scanners, institutions, and treatment protocols, rather than solely on peak performance metrics (93). Practical approaches typically use inputs already part of standard care, such as baseline MRI, amino acid PET when available, key clinical variables, and select blood biomarkers (**Figure 2**). These models deliver outputs that directly influence clinical decisions, including risk groupings, calibrated probabilities, and personalized survival forecasts (94). Critical clinical applications include decision-support tools for prognosis, AI-supported adaptive radiotherapy, and spatial recurrence maps to inform surgical targeting and radiotherapy margins.

As mentioned, monitoring treatment is especially crucial because post-therapy MRI results can be ambiguous. Models capable of distinguishing actual progression from pseudoprogression, preferably using clear clinical endpoints or tissue confirmation, tackle a frequent and vital clinical challenge. For effective implementation, models should incorporate uncertainty estimates, quality-control checks for segmentation and imaging artifacts, and be robust to missing data, often achieved through intermediate or late data fusion techniques (19, 95). Standardized preprocessing, harmonized blood-test handling, external validation, and continuous performance monitoring are essential to ensure the safe and effective operation of clinical decision-support tools (96).

### 3.8 Limitations and barriers to clinical translation

Despite advances, several limitations restrict the clinical application of multimodal AI in brain tumor treatment. Many studies are retrospective, conducted at single centers, underpowered, lack external validation, and have inconsistent ground truth for endpoints such as pseudoprogression versus true progression. Furthermore, variability in imaging across scanners, sequences, and protocols can adversely affect radiomics and DL unless data are harmonized and standardized. Liquid biomarkers are also influenced by handling procedures, assay variations, and biological factors, such as low tumor shedding and blood–brain barrier effects, which reduce their sensitivity and reproducibility. Multimodal models often encounter missing data, such as unavailable PET scans or incomplete blood panels, which can introduce bias if not properly addressed. Additionally, problems such as limited interpretability, calibration errors, and performance drift raise safety concerns, underscoring the need for uncertainty estimates, quality assurance, and ongoing oversight (94, 97). Another key barrier to clinical translation is the changing regulatory landscape for AI-based medical devices. Regulatory agencies such as the U.S. Food and Drug Administration (FDA) and the European Medicines Agency (EMA) are developing frameworks to assess AI-driven clinical decision support systems, particularly those that combine multimodal data (98). Unique challenges include model adaptability over time, dataset transparency, algorithmic bias, and the validation of systems that learn continuously. Multimodal AI systems add regulatory complexity because they combine different data types, such as imaging, clinical variables, and liquid biomarkers, and require evidence of safety, robustness, and generalizability across various clinical settings (99). Addressing these regulatory needs is crucial for moving multimodal AI from research into routine neuro-oncology practice.

### 3.9 Future directions

Future efforts should focus on conducting multi-institutional validation studies that follow standardized imaging protocols, collect biomarker data, and use clinically relevant endpoints aligned with neuro-oncology workflows. Creating benchmark datasets and establishing shared reporting standards for multimodal AI, including metrics for calibration, uncertainty, and failure detection, will enhance reproducibility and enable more rigorous comparisons across studies. Key immediate objectives are to distinguish true tumor progression from pseudoprogression, detect recurrences early, and inform risk-based treatment decisions using MRI/PET and liquid biomarkers. Methodologically, emphasis should be placed on intermediate- and late-fusion techniques that effectively address missing data, modality dropout, and domain shift, and provide transparent explanations of model decisions. Ultimately, multimodal AI has the potential to deliver more accurate, personalized neuro-oncology care by characterizing tumor features, systemic biology, and treatment responses.

## 4 Conclusion

In summary, artificial intelligence is transforming glioma management by facilitating quantitative, multimodal analysis of MRI and PET scans, enabling noninvasive detection of key molecular features, and enhancing outcome predictions. When combined with hematologic and liquid biomarkers such as inflammatory mediators, extracellular vesicle contents, and circulating nucleic acids, AI models can integrate spatial and systemic information about glioma biology that imaging or blood tests alone cannot provide. Achieving real-world impact will require standardized data-collection procedures, rigorous external validation, uncertainty-aware decision-support systems, and ongoing performance monitoring across diverse clinical environments. Collectively, these developments position multimodal AI as a valuable tool for more precise, noninvasive diagnosis, risk assessment, and longitudinal monitoring in glioma treatment.
